# Abrasive, Silica Phytoliths and the Evolution of Thick Molar Enamel in Primates, with Implications for the Diet of *Paranthropus boisei*

**DOI:** 10.1371/journal.pone.0028379

**Published:** 2011-12-07

**Authors:** Diana Rabenold, Osbjorn M. Pearson

**Affiliations:** 1 Department of Anthropology, University of New Mexico, Albuquerque, New Mexico, United States of America; 2 Department of Anthropology, University of New Mexico, Albuquerque, New Mexico, United States of America; University of Arkansas, United States of America

## Abstract

**Background:**

Primates—including fossil species of apes and hominins—show variation in their degree of molar enamel thickness, a trait long thought to reflect a diet of hard or tough foods. The early hominins demonstrated molar enamel thickness of moderate to extreme degrees, which suggested to most researchers that they ate hard foods obtained on or near the ground, such as nuts, seeds, tubers, and roots. We propose an alternative hypothesis—that the amount of phytoliths in foods correlates with the evolution of thick molar enamel in primates, although this effect is constrained by a species' degree of folivory.

**Methodology/Principal Findings:**

From a combination of dietary data and evidence for the levels of phytoliths in plant families in the literature, we calculated the percentage of plant foods rich in phytoliths in the diets of twelve extant primates with wide variation in their molar enamel thickness. Additional dietary data from the literature provided the percentage of each primate's diet made up of plants and of leaves. A statistical analysis of these variables showed that the amount of abrasive silica phytoliths in the diets of our sample primates correlated positively with the thickness of their molar enamel, constrained by the amount of leaves in their diet (R^2^ = 0.875; p<.0006).

**Conclusions/Significance:**

The need to resist abrasion from phytoliths appears to be a key selective force behind the evolution of thick molar enamel in primates. The extreme molar enamel thickness of the teeth of the East African hominin *Paranthropus boisei*, long thought to suggest a diet comprising predominantly hard objects, instead appears to indicate a diet with plants high in abrasive silica phytoliths.

## Introduction

Few dental traits have elicited more interest in the study of human origins and evolution over the past several decades than that of molar enamel thickness in primates. Variation in thickness occurs not only in extant primates, but in Miocene apes and early hominins as well, culminating in the thick enamel of our own genus, *Homo*. Indeed, the observed trend toward thicker enamel is currently considered one of the signature characteristics of hominin evolution [1]–[6]. Early researchers suggested a correlation between molar enamel thickness, open habitats, and terrestriality [7], [8]. Researchers proposed that hominins foraged for hard objects like nuts, seeds, and underground storage organs on, in, or near the ground of the open savanna [9], [10]. As the hominin with the thickest molar enamel, the Plio-Pleistocene East African hominin *Paranthropus boisei* has been considered the ultimate consumer of hard objects [11].

In 1981 these ideas were systematically addressed for the first time in a study measuring and comparing molar enamel thickness in 37 species of Old World monkeys [12]. Kay demonstrated that thick molar enamel did ***not*** correlate with terrestriality. He then proposed that thick enamel was an adaptation for eating hard foods, since primates with thick enamel frequently eat nuts and seeds. While the evidence supporting a correlation between hard foods and molar enamel thickness was largely anecdotal, Kay's hard object feeding hypothesis became widely accepted [13]–[16]. The strongest study testing the hard object feeding hypothesis came in 2008, when researchers recognized that in the twenty-five years since Kay's paper, little hard data had been gathered on the topic [17]. Chimpanzees, with thin enamel, and orangutans, with thicker enamel, were selected for study. Samples of food consumed by both species were collected *in situ* in their respective habitats and tested for hardness by a portable field testing unit. Vogel and colleagues' results showed that orangutans consume harder foods overall than do chimpanzees. More recently, researchers have proposed a dual functional purpose for thick enamel. For primates whose diets contain significant amounts of “small hard objects”—defined as between 5–50 µm in size, such as phytoliths and grit—thick enamel is proposed to resist abrasion. For primates whose diets contain significant amounts of “large hard objects”—defined as those between 2–20 mm in size, such as hard seeds and nuts—thick enamel is proposed to assist in preventing catastrophic fracture of the tooth [18], [19]. The authors put forward evidence from models as well as calculations based on fracture and deformation mechanics to support this hypothesis. Their model can be tested further by greatly expanding the existing database on hardness values for foods consumed by a large number of primate species. With sufficient data the possible role of molar enamel thickness in the consumption of large hard objects should become more evident. It is important for us to note that our paper does not test the hard object feeding hypothesis directly, nor does it show that thick molar enamel cannot be related to hard object feeding.

With respect to hominins, results from recent dental microwear studies of the East African species *Australopithecus anamensis*, *Australopithecus afarensis*, and *Paranthropus boisei* showed patterns of microwear that were not consistent with the heavily scratched and pitted features associated with hard object feeding [6], [20]–[22]. For *P. boisei*, Ungar and colleagues [6] reported only light microwear dominated by fine scratches in seven specimens. Microwear texture analysis of two additional molar specimens of *P. boisei* recently reported from Olduvai Gorge, are also consistent with the lack of features associated with hard object feeding [23]. These results, for a hominin with the thickest molar enamel known, have cast serious doubts on the hard object hypothesis.

These authors make several suggestions to account for the lack of microwear features consistent with hard or even tough foods in the diet of *P. boisei*. They suggest that *P. boisei* may have had a novel diet unlike that of any primate known to date. Another possibility is that *P. boisei* may have consumed tough foods, but its very flat teeth may not have constrained its masticatory movements in an analogous way to the teeth of primates with high shearing crests. This may have resulted in a grinding motion that produced microwear not typical for mastication of tough foods [22]. Cerling and colleagues (2011) reference this same “dentognathic morphology” hypothesis as a possible explanation for the differences between the microwear features of *P. boisei*, whose diet they propose consisted predominantly of grasses, and those of the extant baboon *Theropithecus gelada*, a known grazer [24]. Finally, the authors suggest that the dental microwear examined in the small sample of *P. boisei* molars [now n = 9] may reflect its primary diet, but not its fallback diet, one that may have consisted of harder or tougher food items infrequently consumed during times of resource stress, requiring specialized morphologies [6]. Others argue that after examination of dental microwear from twenty-nine [now n = 31] molar specimens of non-*Homo* East African hominins has failed to show any evidence for hard object feeding, it is perhaps more parsimonious to conclude that they simply did not consume hard objects [25].

### An alternative to the hard object feeding hypothesis

We propose an alternative hypothesis to that of hard object feeding—that the amount of phytoliths in foods correlates with the evolution of thick molar enamel, although this effect is constrained by a species' degree of folivory. The thickness of molar enamel should correlate positively with the amount of phytolith-abundant foods in the diet, but negatively with the percentage of leaves in the diet. The constraint exerted by the percentage of leaves in primate diets is proposed to result from the molar morphology best adapted to prepare leaves for digestion. Leaf-eating primates (folivores) have molars characterized by developed shearing crests, while predominantly fruit-eating species (frugivores) have low, more rounded (bunodont) molar cusps [26]. Folivores also have thinner enamel than frugivores. The correlation among thinner enamel, developed shearing crests and leaf-eating has been attributed to the need for folivores to slice and shred leaves efficiently [16]. Thin enamel more quickly exposes the softer dentine at the cusp apices, resulting in more sharp-edged cusps that maintain greater shearing ability even as the tooth wears [27]. Shredding leaves is important, as it is thought to assist in the energetically costly digestion of their high cellulose content [28], [29].

Our hypothesis stems from our perception that the importance of phytoliths in primates' foods has been under-appreciated. Phytoliths are microscopic mineralized bodies formed within and between the cells of higher plants. Here the term specifically refers to silica phytoliths formed when roots absorb soluble silica in groundwater and transport it into the upper parts of plants [30]. Environmental factors cause some variation in phytolith production, but the leading predictor of the rate of phytolith production in plants is their taxonomic classification. Plants known for high phytolith production accumulate silica in their tissues at high rates wherever they are grown [31]. Deposition sites of phytoliths can vary among plant parts: the leaf may contain a substantial amount of phytoliths, for example, but few in the seed [32]. While leaves are often the plant part in which phytoliths are most abundant *relative to the accumulation of silica phytoliths in that family*, this is not to say that leaves generally are very abundant in phytoliths [33], [34]. The vast majority of dicotyledon families that produce leaves eaten by folivorous primates are not abundant in phytoliths. A common assumption has been that monocotyledons, which include the grass family, are high producers of phytoliths, whereas dicotyledons (eudicots) produce low levels of phytoliths. However, the cumulative evidence from phytolith research shows that while many monocotyledons are indeed high phytolith producers, other monocotyledons produce low levels or none at all. Conversely, a number of dicotyledon families produce substantial levels of phytoliths [33].

While initial phytolith production studies focused predominantly on the quantity of phytoliths found in leaves, more recent studies of the past two decades have added considerable information on the presence of phytoliths in the reproductive parts of both nongrass monocotyledons and a range of dicotyledons [33], [34]. Piperno conducted a study of the reproductive parts of 254 species from over 50 plant families and found that a number of species accumulate phytoliths in their reproductive structures, such as fruits, seeds, and flowers. While these results were preliminary (phytolith abundance was estimated rather than quantified and sample sizes were small), they revealed a pattern of phytolith production related to the abundance of phytoliths in leaves. Species belonging to families found to accumulate abundant phytoliths in their leaves also sometimes, but not always, produce abundant phytoliths in their reproductive parts. Conversely, species in families that do not accumulate abundant phytoliths in their leaves tend not to produce phytoliths in their reproductive structures [35]. Many plant families that produce foods often eaten by primates were found to contain abundant phytoliths both in their leaves and reproductive parts, such as the Arecaceae (palms), Marantaceae, Musaceae, Boraginaceae, Burseraceae, Chrysobalanaceae, Dilleniaceae, Moraceae, Ulmaceae, and Urticaceae families [34], [35].

### The abrasiveness of silica phytoliths

Phytoliths are very hard small objects, although how hard has recently become a matter of some debate. While there is some evidence that dietary intake of particles softer than tooth enamel can cause wear in teeth [36], [37], most researchers attribute abrasive dental wear to contact with materials as hard, or harder, than tooth enamel. By this definition, three sources of dental abrasion have been identified: tooth enamel itself, in tooth-to-tooth contact during mastication of soft or small foods; exogenous grit or dust ingested from plants or soils; and silica phytoliths from plants [16], [38], [39].

In 1959 Baker and colleagues conducted two types of hardness tests on silica phytoliths and sheep tooth enamel. The Mohs hardness test is a simple method of characterizing the relative (not proportional) scratch resistance of various minerals through the ability of a harder material to scratch a softer material, on a scale of 1–10, with 1 representing the softest and 10 the hardest material [40]. Baker and colleagues' results for the Mohs hardness test on sheep enamel gave a range of 4.5–5.0. A sample of opal, the mineraloid equivalent of amorphous silicon dioxide as a proxy for silica phytoliths, gave a Mohs value of 5.5–6.5 [41]. These values are in agreement with standard published Mohs values for both tooth enamel (5) and amorphous silica (5.5–6.5) [42].

The second hardness test measured the resistance of a material to indentation under a given load using a diamond indenter. These results were reported in the Knoop hardness scale (HK). The sample of molar sheep tooth enamel gave Knoop values ranging from 270–382, while silica phytoliths extracted from oats (*Avena* spp.) gave Knoop values ranging from 590–610. On the basis of these results, Baker and colleagues concluded that the chief agent of wear in sheep tooth enamel was most probably the presence of silica phytoliths in their diet. They observed abundant silica phytoliths in sheep feces, as well as occasional exogenous particles of quartz. They concluded that hard exogenous particles on the grass or soil probably also contributed to tooth wear.

This 1959 study has served as the key reference supporting the assertion in many later studies that silica phytoliths are principal abrasive agents in dental wear due to their greater hardness than tooth enamel [38], [43]–[47]. A recent study by Sanson and colleagues challenges these assertions. Although they did not conduct Mohs hardness tests, they performed indentation hardness tests on both sheep tooth enamel and silica phytoliths. Their results for sheep enamel, converted to Knoop values from the Vickers hardness scale (HV), ranged from 579–598, very similar to the values obtained by Baker and colleagues [48]. On the other hand, their indentation hardness values for silica phytoliths extracted from four species of grasses, had a maximum Knoop value of 221, a result significantly different from the range of 590–610 obtained by Baker and colleagues. On the basis of these results, the authors concluded that silica phytoliths are softer than tooth enamel. They therefore questioned the role, if any, that silica phytoliths play in tooth enamel abrasion and wear. A serious failing of the Sanson and colleagues' paper is that they did not test for the hardness of any particles of exogenous dust or grit, particularly given that they propose these particles as the chief, and perhaps sole, agents of abrasive wear of tooth enamel. As the authors acknowledge, additional hardness tests comparing silica phytoliths and mammalian tooth enamel are much needed to clarify this important issue.

Despite the contrary evidence of Sanson and colleagues, there are other lines of evidence suggesting that phytoliths are capable of indenting tooth enamel. One comes from high resolution scanning electron (SEM) photographs of silica phytoliths indenting tracks directing into the tooth enamel of hominoids and humans. Thirty phytoliths were found on four tooth specimens of the fossil Pleistocene ape *Gigantopithecus blacki* and SEM photographs clearly show phytoliths at the end of indented tracks in the enamel surface. Lalueza Fox and Pérez-Pérez also photographed phytoliths embedded in the dental enamel of seven teeth from separate individuals from a medieval Spanish site [49]. Two of these are associated with striations on the enamel surface. In a later study, Lalueza Fox and colleagues reported identifying phytoliths from cereal plants on tooth enamel of specimens from a Late Roman necropolis in Tarragona, Spain [38]. Again, SEM photographs showed phytoliths associated with particular scratches on tooth enamel.

An experimental study by Gűgel and colleagues simulated masticatory contact between abrasives in food and tooth enamel. They utilized a device consisting of two wheels that move past one another in different directions, one fitted with twenty previously unerupted, unworn human molar specimens, and the other sliding a mushed sample of food laterally across the teeth [45]. Four different samples of food mush were prepared, each containing only one species of cereal grain. Prior to extraction of the phytoliths from the plant material, the grains were hand washed repeatedly in distilled water and weak hydrochloric acid to remove all exogenous dust and grit. However, particles of mill stone were introduced into the food during the milling process. The abrasiveness of each cereal species was determined by the amount of silica phytoliths each cereal contained, based on the extracted dried silica residues obtained prior to milling. The degree of abrasiveness of each cereal was found to correlate with loss of tooth enamel, as determined by noncontact optical sensor measurements of surface roughness and 3-D laser scans of tooth enamel taken both before the device experiment (baseline), and after 200,000 simulated chewing cycles. The researchers found significant correlations as well between the number and size of microwear pits on the molar specimens, and the species of cereal in each food sample. Indeed, each tested cereal species was found to cause a matching, diagnostic pattern of microwear pitting. While wear caused by abrasive particles of exogenous grit must be considered, it is difficult to understand how characteristic cereal-specific pits could result from abrasion by grit, and not by cereal phytoliths.

The grass family, Poaceae, produces particularly high levels of phytoliths [50], and grazing ungulates show dental adaptations to an abrasive diet in the form of tall-crowned (hypsodont) teeth, [51] which add more enamel volume to resist abrasion from phytoliths [52]. A quantification of silica phytoliths in East African vegetation showed that grasses contained 4.95% percent dry matter (%DM) of silica compared with 0.56–1.46% DM of silica in browse [53]. Analysis of large databases of dental microwear in ungulates demonstrate significant differences in dental microwear scratch sizes and densities between grass consumers and nongrass consumers, with significantly greater scratch densities for grazers [54], [55]. While greater scratch density for grazers may indicate larger amounts of grit or dust on ground vegetation in open habitats, significant differences in scratch sizes between the two groups is not a result consistent with exogenous grit as the chief or sole abrasive agent.

While some tooth abrasion may be caused by the silica contained in exogenous abrasives, especially in more open habitats [52], [56], there is evidence that phytoliths may be the principal abrasive agents even in these environments. In a study of two sympatric species of hyraxes, Walker and colleagues showed that the teeth of the predominantly grazing hyrax (dry season diet, 57% grass; wet season diet, 78% grass) demonstrated far greater wear, especially during the wet season, than the browsing species that ate predominantly leaves year round [57]. Fecal analysis showed significantly greater abundance of phytoliths in pellets from the grazing hyrax, while dust particles were found in equal amounts in the feces of both species. The preponderance of evidence suggests a crucial role of phytoliths in producing tooth wear.

## Methods

To test the hypothesis that the abundance of phytoliths and proportion of leaves in the diet determine enamel thickness, we selected twelve primates with known values for standardized molar enamel thickness and whose proportional consumption of plant food by species and/or family could be gleaned from the literature (Table 1). The sample includes primates with a wide variation of molar enamel thickness, including those with the thickest molar enamel. Nevertheless, our sample lacks any representation of anthropoids that are dedicated folivores. This omission is unfortunate, but results from the absence of any published values for relative enamel thickness (see below) for members of this group for which we had the requisite dietary data. However, all three of the sampled subspecies of gorilla show percentages of leaves consumed ranging from 45.69 to 51.3, with a mean of 49.16. These values are comparable to those observed in leaf-eating anthropoids such as *Alouatta palliata* (43.5% [29]; 48% [58]; 53.7% [59]; 51.5% [60]; *Colobus badius* (39.9% if leaf buds are not counted, 54.4% if leaf buds are counted) [61]; *Colobus satanas* (37.8%) [61]; *Colobus guereza* (52.75% mean for 2 groups) [62]; and *Presbytis thomas* (48%) [63].

**Table 1:** Dietary data and RETs.

Primate species	No. of studies	RET value	Phytolith Load A (%)	Phytolith Load B (%)	Leaves in total diet (%)	Identified plants in diet (%)	Plants not scored^1^ (%)
*Daubentonia madagascariensis*	2	21.68	71.83	49.13	0.0	69.8	0.0
*Cebus apella*	3	19.57	72.31	61.07	0.0	82.1	7.27
*Lophocebus albigena*	5	16.85	49.43	36.7	3.9	84.89	19.46
*Papio cynocephalus*	2	16.11	77.43	77.02	2.4	94.79	13.47
*Pongo pygmaeus*	4	15.33	49.25	40.32	15.65	63.95	26.28
*Cebus capucinus*	3	15.13	51.07	39.38	1.97	69.34	19.41
*Pan paniscus*	2	14.0	83.57	82.31	18.2	93.59	2.33
*Cercocebus torquatus*	2	12.89	40.59	38.11	5.74	96.97	19.19
*Pan troglodytes*	7	11.60	70.79	68.45	17.47	87.79	10.0
*Hylobates lar*	3	11.09	59.31	54.46	18.57	72.31	21.79
*Gorilla spp.*	3	9.66	49.3	47.08	49.16	87.79	25,51
*Chiropotes satanas*	3	9.54	33.26	32.05	1.72	96.77	47.68

Summary of the dietary variables and relative molar enamel thickness (RET) values, arranged in the order of the largest to smallest), for each primate species in the sample.

1 Percentage of plants in each primate species not categorized for phytolith abundance due to lack of sufficient information.

For each species in our sample we examined the literature for detailed dietary information, including comprehensive food lists, feeding behavior, plant parts eaten, percentage of the diet made up of plants, and proportional percentages of each plant species or family eaten. Wherever possible, we used multiple studies representing different habitats within each primate's range (see Text S1, Table S1).

General levels of phytoliths present in each diet were determined by scoring each plant species consumed according to categories developed by Piperno in her comprehensive summary of research on the levels of phytolith production in plant families [33]. Piperno divided phytolith abundance into three general categories: (1) often common to abundant; (2) often uncommon to rare or absent; and (3) not observed. The highest category of production, “often common to abundant,” is defined as a plant family in which “a great many species…usually well over 50% of the total studied, produce significant amounts of phytoliths, that when expressed as a percentage of dry plant weight would approach, equal, or be greater than the 2–5% values commonly reported for grasses” (p. 22) [33]. The total percentage of a primate's diet made up of foods from plant families in the “often common to abundant” category was calculated for each study. No other category was scored as contributing to this total, even though other families may have contained phytoliths. Nectar, even when coming from a plant family rich in phytoliths, was not counted, as it was judged to be swallowed rather than masticated. In a few cases in her Tables 2.2, 2.3, and 2.4, Piperno [33] presented quantified data indicating that a particular plant species, or plant part, consumed by a primate in our sample is an exception to the general category of phytolith production for the family (e.g., maize kernels have fewer phytoliths than expected for Poaceae/Graminae). In these cases, the scoring of phytolith abundance for those items was adjusted accordingly. Nevertheless, such precise data were available for relatively few plants. We should note that a number of plant species in each dietary study could not be scored due to lack of sufficient information regarding their phytolith abundance (Table 1).

**Table 2:** Multiple regressions on raw data.

Term	Estimate	Std Error	t Ratio	Prob>|t|
Intercept	7.414629	1.986977	3.73	0.0058
Phytolith load A	0.4515076	0.081589	5.53	0.0006
Phytolith load B	−0.357871	0.078073	−4.58	0.0018
% leaves eaten	−0.081616	0.037286	−2.19	0.0600

R^2^ = 0.8715, p<0.0006.

RET = 7.4146+0.4515 (Phyto_A)−0.3579(Phyto_B)−0.0816(%_leaves)±1.5920. Parameter Estimates.

**Figure 1:**
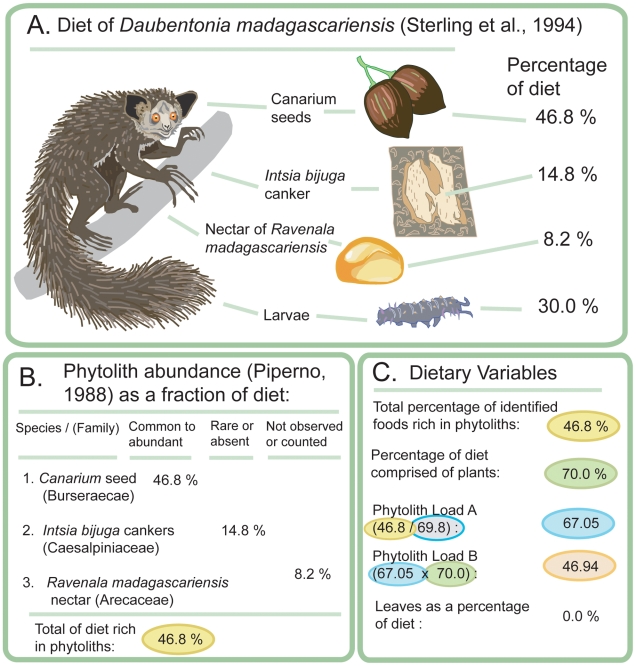
Steps in development of variables from raw data obtained from one dietary study [**53**]. (The aye-aye is modified, with permission, from an illustration by Stephen D. Nash). **Panel A**: Percentage of feeding time spent on each food consumed by *Daubentonia madagascariensis* in direct observations taken from one dietary study. **Panel B**: The phytolith abundance of each of the plant foods is determined by categories developed by Piperno [33], summarizing research of phytolith abundance in plants. Only the dietary percentage of plants categorized as “common to abundant” in phytoliths was totaled. In this study, the total percentage of feeding time spent on identified plant foods rich in phytoliths is 46.8. **Panel C**: How the three variables in this study were obtained. The total percentage of identified foods rich in phytoliths, as shown in Panel B, is 46.8. The percentage of the diet comprised of plants is 70.0. The percentage of ***identified*** plant foods is 69.8. Phytolith Load A is calculated as the percentage of identified foods rich in phytoliths (46.8) divided by the percentage of identified plant foods (69.8), or 67.05%. Phytolith Load B is calculated by multiplying Phytolith Load A by the percentage of the diet made of plants: 67.05×70.0 = 46.94%. The third variable consists of leaves as a percentage of the total diet.

The following three variables were then calculated for each dietary study: (1) Phytolith Load A; (2) Phytolith Load B; and (3) the percentage of the total diet composed of leaves. Phytolith Load A represents the sum of all observed feeding minutes or bouts spent by a primate on each plant species belonging to a plant family categorized as “often common to abundant in phytolith production” (p. 22) [33], as a proportion of plant foods identified to the family level in the diet. If a plant was not identified to the family level, it could not be scored for phytolith abundance. While Phytolith Load A is a key variable, it does not capture variation regarding the proportion of diet made up of plant foods. Phytolith Load B is therefore a separate variable that expresses that proportion. It is calculated by multiplying Phytolith Load A by the percentage of the total diet made up of plant foods. While most primates in our sample had diets almost totally dominated by plants, in which case their Phytolith Load A and Phytolith Load B values are very similar, even identical in cases where 100% of the diet was made up of plants, some had significant proportions of non-plant foods in their diets. The inclusion of both the Phytolith Load A and Phytolith Load B variables allowed certain trends to be detected in the statistical analysis that would not have been noted otherwise. The final variable—percentage of leaves in the total diet—is self-explanatory. Figure 1 illustrates the steps we took to convert the raw data from a dietary study into the variables used in the statistical analysis. The data from multiple study sites were averaged to obtain the phytolith loads and leaf percentage for each species (Table 1).

Enamel thickness was gauged by published two-dimensional “relative enamel thickness” (RET) values [2], based on measurements taken from thin sections through the tips of the mesial molar cusps viewed under a scanning electron microscope (see Text S2). Both physical 2-D sections and comparable 3-D virtual sections using microcomputed tomography (mCT) and synchrotron microtomography (SR-mCT) have been shown to provide measurements of the thickness of enamel over the molar mesial cusps that are in broad general agreement with one another across species [64]–[66]. We selected 2-D physically sectioned RETs over virtual 3-D RETs, as more of the former were available in the literature for extant primates whose published dietary data also met the specific requirements of our study. Some researchers utilizing 3-D tomographic techniques criticize the 2-D physical sectioning methodology for its inability to capture the distribution of enamel over the entire crown, thereby placing exaggerated importance on enamel thickness over the cusp apices [67]. However, the thickness of enamel over the cusps is of particular interest to us, due to the role of folivory in our hypothesis.

## Results

**Figure 2:**
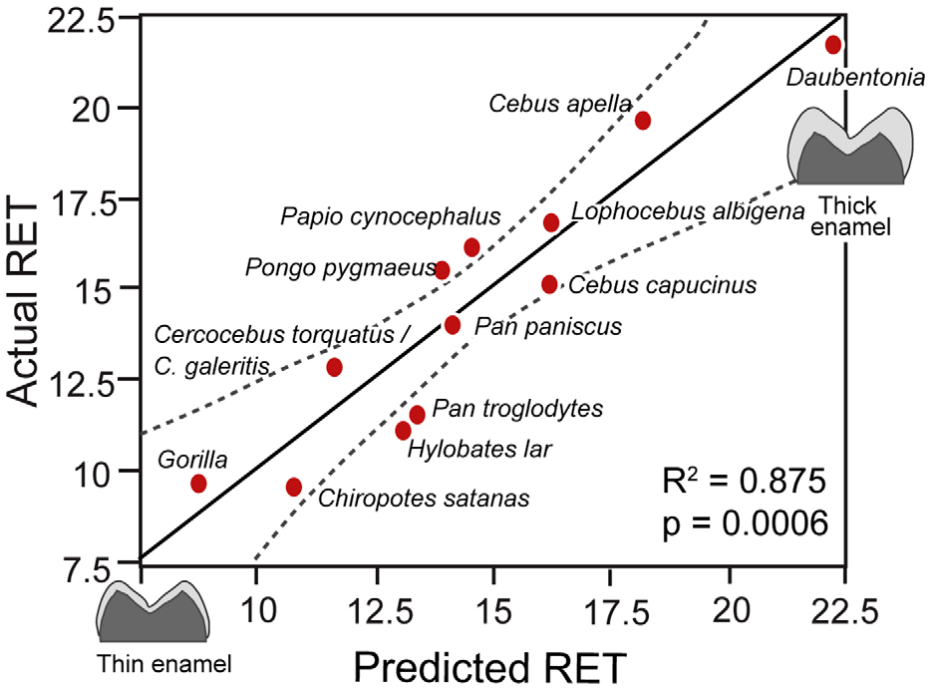
Predicted versus actual values of relative enamel thickness (RET) based on the raw data for three dietary variables.

We used JMP 6.0.3 to calculate pair-wise correlations between variables as well as a series of multiple regressions to predict RET from the other variables. Pair-wise correlations showed that only percentage of leaves eaten (%_leaves) correlated significantly with RET (r = 0.586, p = 0.045). Phytolith load A (Phytolith_A) also showed a modest, but non-significant association with RET (r = 0.467, p = 0.126). Values for Phytolith_A and phytolith load B (Phytolith_B) were strongly associated (r = 0.915, p<0.0001). Phytolith Load B showed a non-significant association with RET (r = 0.1205, p = 0.7091) (Table S6). A multiple regression to predict RET from %_leaves, Phytolith_A, and Phytolith_B indicated that RET was highly predictable from these variables (R^2^ = 0.875, p<0.0006) (Figure 2). In the model, both measures of phytolith loads exerted stronger effects than the percentage of leaves eaten (Table 2).

Analysis of trends in related species is problematic because closely related taxa are not strictly independent data points due to their shared evolutionary history [68]. To address this problem the data were transformed into eleven phylogenetically independent contrasts and later scaled by branch length, the square root of the time separating the nodes [68], [69]. Estimates from Perelman and colleagues' synthesis of primate phylogeny were used for branch lengths [70]. Phylogenetic contrasts unscaled by branch length also showed a strong relationship between RET and the dietary variables (R^2^ = 0.764, p = 0.0134). Adjustment by branch length reduced the strength of these relationships although the model remained significant at p<0.05 (R^2^ = 0.661, p<0.0453). Branch length weights are moderately correlated with contrasts in RET (r = 0.654, p = 0.0291), but weakly correlated with contrasts in the dietary variables (−0.106≤r≤0.200) (Figure S1; Tables S2, S3, S4, S5, S6, S7, S8).

## Discussion

Analysis of the data shows that RET appears to be strongly related to phytoliths and, to an extent, also to percentage of leaves eaten. This is not to say that many primates with thick enamel do not also consume hard objects. *Cebus apella* is a known hard object feeder, capable of fracturing even the hard fruits of the *Astrocaryum* palm with its molars, as well as exploiting other palm parts such as the tough tissues of palm pith and meristems [71]. A striking overlap exists between primates who exploit palms and those with thick molar enamel. All four extant primates with the highest known molar RETs (see Table 1) exploit palm fruits: *Daubentonia madagascariensis* [72]; *Cebus apella* [71]; *Lophocebus albigena* [73] and *Papio cynocephalus* [74]. The correlation between palm fruit consumption and thick molar enamel has commonly been attributed to the hardness of these fruits [12]. However, all members of the palm family (Arecaceae) also contain abundant phytoliths in all parts of the plant [33]. The seeds of *Hyphaene petersiana*—a palm genus exploited by *Cercocebus galeritus* and *Papio cynocephalus* [75]—contained 147 million phytoliths per gram of acid insoluble fraction (AIF) [76]. Quantification of phytoliths in 16 species of trees, sedges, grasses, and palms, showed that some palm parts produced the highest levels of phytoliths. By contrast, the leaves of species of *Typha*, a genus of wetland monocotyledonous reeds, had the lowest levels, at 100,000 phytoliths/g AIF [76].

On the basis of its feeding behavior, however, *Daubentonia madagascariensis* (the aye-aye) does not masticate hard palm nuts, or hard objects generally. It gnaws into the exocarp of unripe fruits of the coconut palm with its ever-growing incisors and scoops out the soft, yoghurt-like nutmeat with the claw of its elongated third digit [72]. The aye-aye's diet does contain seeds with hard exocarps, principally *Canarium* spp. and *Terminalia catappa*. However, it uses its incisors to breach these seed coats, extracting bits of the endosperm, again with its elongated digit [77], [78]. Aye-ayes scrape the bark of *Intsia* spp. trees with their incisors to eat the underlying cambial layer, a food described as “tender” (p. 40) [72]. The remaining principal components of the aye-aye's diet are insect larvae and nectar. It is difficult to see what hard objects in its diet could account for its molar RET value, the highest known for any extant primate [13]. The aye-aye also hones its ever-growing incisors to a sharp edge by repeatedly manipulating an abrasive plant part—commonly a slim palm tree or bamboo stem—in the gap between the incisors and the molars [72], [79]. The epidermis of bamboo and palm is very high in phytoliths [33]. As would be predicted by our model, *D. madagascariensis* consumes plant foods high in phytoliths, eats no leaves, and its honing behavior utilizes plant stems high in phytoliths.

In our statistical analysis, 87.5% of variation in the RET values of our sample primates is accounted for by the relationship of these values to phytolith abundance and percentage of leaves in the diet. This accords well with the work of Macho & Spears, who found that thick enamel consistently reduced the tensile stresses to which teeth are subjected—and therefore enhanced the strength of teeth under a given load—by about 15% [80]. They concluded that additional factor(s) must be the principal driver(s) of the evolution of thick enamel, and suggested abrasion resistance could be such a factor. Our results showed that the need to resist abrasion by silica-rich phytoliths appears to be a key selective factor in the evolution of molar enamel thickness in primates. Factors that reduce dental wear have been correlated with increased dietary quality and longevity in mammals, resulting in higher reproductive success [81]–[83].

These results suggest several additional directions for future research. One is the expansion of the sample to include more extant primates. The present sample size was limited primarily by the number of relative enamel thickness values available in the literature. Additional RET studies would allow for a larger sample. Field research is also needed to determine more precise phytolith abundance values for the principal plants and plant parts in primate diets. To control for the confounding variable of the percentage of leaves in the diet, the precise phytolith abundance of the diets of two primates that do not eat leaves but that have molars with contrasting enamel thickness values, should be compared. Our hypothesis predicts that the thin enameled primate's diet will be significantly lower in phytoliths than that of the thick enameled primate.

### Implications for the diet of *Paranthropus boisei*

These results also have important implications for the diets of some early hominins, as the australopithecines are characterized by intermediate to hyper-thick molar enamel [84], [85]. We suggest that australopithecine diets consisted of plant foods high in phytoliths, few if any leaves, and included a substantial component (>50% if no leaves were consumed) of non-plant foods. The equation generated by our model—RET = 7.4146+0.4515 (Phyto_A)−0.3579(Phyto_B)−0.0816(%_leaves)±1.5920 (see Table 2)—indicates that in thick enameled primates, Phytolith Load B must be substantially lower than Phytolith Load A, a pattern that correlates with a decreased percentage of plant foods in the diet. In the model, increased consumption of leaves corresponds to lower values of RET. Extension of the model to *P. boisei* predicts a feeding ecology markedly different from any primate in the sample. There is always a danger of extrapolating beyond the range of a regression model, but an unusual diet may make sense because *P. boisei* also has substantially thicker enamel than any of the species in the comparative sample.

When these dietary inferences are combined with the unusually high C_4_ signatures found in several early hominin diets—evidence that they consumed substantial amounts of plants utilizing the C_4_ photosynthetic pathway such as grasses and sedges, and/or animals that consumed such plants—additional aspects of their diets can be deduced [86]–[88]. This particularly applies to *Paranthropus boisei*, which has both the thickest molar enamel (RET = 34.91) [84] and the highest C_4_ dietary component of any hominin sampled so far. Two recent carbon isotope studies of the molar enamel of *P. boisei* showed a mean C_4_ dietary component of 77% and 79% respectively [89], [24]. Both papers suggest that C_4_ wetland sedges could account for the C_4_ signature, although Cerling and colleagues propose that grass blades comprised the more likely principal plant food for *P. boisei*, largely due to the greater availability of this food resource in the environment [24]. While both sedges and grasses are abundant in phytoliths, we propose that C_4_ sedges are more likely candidates for the predominant portion of *P. boisei*'s plant diet. The flat, low-crowned molar morphology of *P. boisei* evinces the very opposite morphology of tall-crowned cheek teeth with the sharp, shearing edges formed along ridges of complex infolded occlusal enamel that are required to shred leaves of grass, and that characterize all grazers, including the sole higher primate grazer, *Theropithecus gelada* [51], [90]. *P. boisei*'s dentition is consistent with the consumption of plant pith (i.e., parenchymatous ground tissue found in the center of stems) and rhizomes. Pith and roots are eaten by both species of the genus *Pan*, whose teeth are rounded and low-crowned (bunodont), similar to *P. boisei* [91], [92], and by bunodont, thick enameled primates such as *Cebus apella* and *Lophocebus albigena* [93], [94]. The exploitation of the rhizomes of wetland sedges is compatible with the hypothesis put forward by a number of researchers that early hominin diets, including that of *P. boisei*, may have contained a substantial portion of plant underground storage organs (USOs) [10], [95], [96].

As leaves are a valuable source of protein for frugivorous primates [58], [97], *P. boisei* may have increased its intake of protein rich animal foods to compensate for the absence of leaves in its diet, and to subsidize its significant consumption of fibrous, low protein, low fat plant foods [98]. Protein and fat contents for papyrus shows that the edible parts of this plant are poor sources of these nutrients. The protein content of the rhizome of *C. papyrus* is 1.0 grams per 100 grams of wet weight; and the base of the stem (another part of the plant eaten by humans [99]) contains 0.5 g of protein per 100 grams of wet weight [89]. The pith of 1 meter of culm was found to contain 1.7% crude protein by percentage of dry matter, the lowest protein levels found in nutritional analyses of six species of pith commonly consumed by *Pan troglodytes* [100]. With respect to fat content, the fat per gram of the culm is 0.2 per 100 grams of wet weight, and the fat per gram of the rhizome is 1.0 [89]. Both the pith and rhizome of papyrus, however, do supply substantial amounts of energy in the form of carbohydrates [89].

We suggest that the diet of *P. boisei* was very abrasive, but not as abrasive as *T. gelada*'s. Our reasoning is based on two lines of evidence. First, while the sedge family produces a significant number of phytoliths, grasses produce substantially more [33], [76]. The amount of silica in the pith of *Cyperus papyrus* was quantified as 2.26 percent of dry matter (%DM), while the leaf blade of *Cynodon dactylon*, a C_4_ savanna grass found throughout Africa, contained 3.08%DM [101]. The silica content of African grass leaves has been measured at levels as high as 18.03%DM [53]. Second, in our view *T. gelada* exhibits two adaptations for resisting abrasive foods. It has “intermediate/thick” enamel on the occlusal surface of its molars, as classified by the enamel thickness scoring system of Martin [2], [102], with a 2-D RET value of 15.51, slightly higher than that of *Pongo pygmaeus* at 15.33 [13]. In addition, *T. gelada* has tall-crowned teeth [103], [104]. Hypsodont or tall-crowned teeth are an adaptation found in grazing herbivores, in which greater enamel volume is added by raising the height of the crown. This adaptation is most often attributed to the need to resist abrasion from the high levels of silica phytoliths in grasses, and from grit (which also contains silica) found in ground forage in open habitats [51], [52], [105]. The tall-crowned teeth of grazers, including those of *T. gelada*, are accompanied by complex molar occlusal surfaces characterized by sharp ridges or crests of enamel that shred blades of grass in a way analogous to shearing crests in leaf-eating folivores [51].

The cranial morphology of *Paranthropus boisei* features a flat, vertical facial profile (orthognathic), laterally flaring zygomatic bones, pronounced postorbital constriction, and a robust, thick mandibular corpus. These features are commonly ascribed to a suite of hyper-masticatory traits necessary for the fracturing of hard objects [106]. The skull morphology of the fossil Madagascar lemur *Hadropithecus stenognathus* is strikingly similar to that of *P. boisei*'s and, like *P. boisei*, *H. stenognathus* has long been classified as a probable hard object feeder [107]. New research on the biomechanics of this lemur, using digitally reconstructed models of the skull, showed that *H. stenognathus* appears not to have been mechanically adapted for the fracture of hard objects. Rather, a diet comprising large amounts of fibrous plant foods requiring a great deal of mastication is indicated [108]. The mechanics of repetitive chewing of tough foods such as plant pith and rhizomes, as we propose for *P. boisei*, is consistent with these findings.

In order to maintain a C_4_ signature as high as those shown by recent studies, *P. boisei* would have had to live in an ecosystem that provided abundant edible C_4_ foods year round. Apart from C_4_ grasses, edible C_4_ plant foods are rare in tropical Africa, and are found primarily in wetlands [109]. Most habitats in which edible C_4_ plants would be present, would also contain much greater numbers of edible C_3_ plants. The exception, as Peters & Vogel note, would be “a vast marsh, dominated by papyrus” (p. 225) [109]. Papyrus is a C_4_ giant wetland sedge whose pith is known to be consumed by chimpanzees [100], and whose pith and rhizomes are consumed by present-day humans [110]. Papyrus is often found in vast, monotypic swamps that dominate the permanent freshwater wetlands of present-day Africa in geomorphological and hydrological contexts often associated with the paleoenvironments of *P. boisei* [111]–[113]. We therefore suggest that the plant component of *P. boisei*'s diet comprised significant amounts of the pith and possibly rhizomes of wetland sedges, and other aquatic plants with C_4_ or C_4_-like signatures [114] that grew in a freshwater tropical marsh dominated by papyrus. The recently published oxygen isotope values for *P. boisei* are consistent with a highly water-dependent animal [24], [115]. Many animals in a C_4_-plant-dominated environment would also likely have had significant C_4_ signatures, and would have contributed to *P. boisei*'s total C_4_ dietary component.

## Supporting Information

Text S1 **Relative enamel thickness (RET) values.** Additional information on the methodology used in the literature to obtain these values.
(DOC)

Text S2 **Dietary Criteria.** Additional information on the criteria used to select the primate species used in our sample.
(DOC)

Figure S1 **Phylogeny of the species included in the study with ages of splits indicated in millions of years (data from Perelman et al., 2011** [**70**] **).** The phylogeny (Figure S1) of the twelve primate species was transformed into eleven phylogentically independent contrasts following Garland et al.'s [69] application of Felsenstein's method [68]. The phylogenetically independent contrasts used in some of the analyses are as follows: Contrast 1, *Cebus apella* versus *Cebus capucinus*; Contrast 2, *Chiropotes* versus the *Cebus* clade; Contrast 3, *Pan paniscus* versus *Pan troglodytes*; Contrast 4, *Gorilla* versus the *Pan-Homo* clade; Contrast 5, *Pongo* versus the African ape clade; Contrast 6, *Hylobates lar* versus the great ape clade; Contrast 7, *Lophoccebus albigena* versus *Papio cynocephalus*; Contrast 8, Clade of *Lophocebus* plus *Papio* versus the amalgam of *Cercocebus torquatus/C. galeritis*; Contrast 9, Old World monkeys versus Hominoids; Contrast 10, Platyrrhines versus Catarrhines; Contrast 11, *Daubentonia madagascariensis* versus Anthropoids.
(TIF)

Table S1 **Dietary studies from which data was obtained for each primate species in the sample, along with sources of their RET values.**
(DOC)

Table S2 **Transformed data for the phylogenetically independent contrasts.**
(DOC)

Table S3 **Multiple regression on raw data to predict RET from dietary variables.**
(DOC)

Table S4 **Multiple regression on data transformed into phylogenetically independent contrasts.** These contrasts predict the difference in RET from differences in the dietary variables, unscaled by branch length (time).
(DOC)

Table S5 **Multiple regression on data transformed into phylogenetically independent contrasts, scaled by time.**
(DOC)

Table S6 **Correlations between the raw variables.**
(DOC)

Table S7 **Correlations between variables transformed into phylogenetically independent contrasts, unscaled by time.**
(DOC)

Table S8 **Correlations between variables transformed into phylogenetically independent contrasts scaled by time.**
(DOC)

## References

[1] Gantt DG (1977). Enamel of primate teeth: its thickness and structure with reference to functional and phyletic implications [PhD].

[2] Martin LB (1985). Significance of enamel thickness in hominoid evolution.. Nature.

[3] Andrews P, Martin LB (1991). Hominoid dietary evolution.. Phil Trans R Soc Lond B.

[4] White FD, Suwa G, Asfaw B (1994). *Australopithecus ramidus*, a new species of early hominid from Aramis, Ethiopia.. Nature.

[5] Grine FE (2004). Geographic variation in tooth enamel thickness does not support Neandertal involvement in the ancestry of modern Europeans.. S Afr J Sci.

[6] Ungar PS, Grine FE, Teaford MF (2008). Dental microwear and diet of the Plio-Pleistocene hominin *Paranthropus boisei*.. PLoS ONE.

[7] Jolly CJ (1970). The Seed-Eaters: A New Model of Hominid Differentiation Based on a Baboon Analogy.. Man New Series.

[8] Leakey MG, Feibel CS, McDougall I, Walker A (1995). New four-million-year-old hominid species from Kanapoi and Allia Bay, Kenya.. Nature.

[9] Andrews P, Walker A, Isaac GL, McCown ER (1976). The Primate and Other Fauna from Fort Ternan, Kenya.. Human Origins: Louis Leakey and the East African Evidence.

[10] Laden G, Wrangham RW (2005). The rise of the hominids as an adaptive shift in fallback foods: Plant underground storage organs (USOs) and australopith origins.. J Hum Evol.

[11] Wood B, Constantino P (2007). *Paranthropus boisei*: Fifty years of evidence and analysis.. Yrbk Phys Anthropol.

[12] Kay RF (1981). The Nut-Crackers—A New Theory of the Adaptations of the Ramapithecinae.. Am J Phys Anthropol.

[13] Shellis RP, Beynon AD, Reid DJ, Hiiemae KM (1998). Variations in molar enamel thickness among primates.. J Hum Evol.

[14] Schwartz GT (2000). Taxonomic and functional aspects of the patterning of enamel thickness distribution in extant large-bodied hominoids.. Am J Phys Anthropol.

[15] Lambert JE, Chapman CA, Wrangham RW, Conklin-Brittain NL (2004). Hardness of Cercopithecine foods: implications for the critical function of enamel thickness in exploiting fallback foods.. Am J Phys Anthropol.

[16] Lucas PW (2004). Dental Functional Morphology.

[17] Vogel ER, van Woerden JT, Lucas PW, Atmoko SSU, van Schaik CP (2008). Functional ecology and evolution of hominoid molar enamel thickness: *Pan troglodytes schweinfurthii* and *Pongo pygmaeus wurmbii*.. J Hum Evol.

[18] Lucas P, Constantino P, Wood B, Lawn B (2008). Dental enamel as a dietary indicator in mammals.. BioEssays.

[19] Lucas PW, Constantino PJ, Chalk J, Ziscovici C, Wright BW (2009). Indentation as a Technique to Assess the Mechanical Properties of Fallback Foods.. Am J Phys Anthropol.

[20] Grine FE, Ungar PS, Teaford MF, El-Zaatari S (2006a). Molar microwear in *Praeanthropus afarensis*: Evidence for dietary stasis through time and under diverse paleoecological conditions.. J Hum Evol.

[21] Grine FE, Ungar PS, Teaford MF (2006b). Was the Early Pliocene hominin “*Australopithecus*” *anamensis* a hard object feeder?. S Afr J Sci.

[22] Ungar PS, Scott RS, Grine FE, Teaford MF (2010b). Molar microwear textures and the diets of *Australopithecus anamensis* and *Australopithecus afarensis*.. Phil Trans R Soc Lond B.

[23] Ungar PS, Krueger KL, Blumenschine RJ, Njau JK, Scott RS (2011). Dental microwear texture analysis of hominins recovered by the Olduvai Landscape Paleoanthropology Project, 1995–2007.. J Hum Evol.

[24] Cerling TE, Mbua E, Kirera FM, Manthi FK, Grine FE (2011). Diet of *Paranthropus boisei* in the early Pleistocene of East Africa.. Proc Natl Acad Sci U S A.

[25] Sponheimer M, Codron D, Passey BH, de Ruiter D, Cerling TE (2009). Using carbon isotopes to track dietary change in modern, historical, and ancient primates.. Am J Phys Anthropol.

[26] Fleagle JG (1999). Primate Adaptation and Evolution.

[27] Ungar PS, M'Kirera F (2003). A solution to the worn tooth conundrum in primate functional anatomy.. Proc Natl Acad Sci U S A.

[28] Rosenberger AL, Kinzey WG (1976). Functional patterns of molar occlusion in platyrrhine primates.. Am J Phys Anthropol.

[29] Milton K, Montgomery GG (1978). Behavioral Adaptations to Leaf-eating by the Mantled Howler Monkey.. The Ecology of Arboreal Folivores.

[30] Jones LHP, Handreck KA (1967). Silica in soils, plants, and animals.. Advances in Agronomy.

[31] Metcalfe CR, Chalk L (1950). Anatomy of the Dicotyledons. 2 vols.

[32] Piperno DR (1985a). Phytolith analysis and tropical paleo-ecology: Production and taxonomic significance of siliceous forms in New World plant domesticates and wild species.. Rev Palaeobot and Palynol.

[33] Piperno DR (1988). Phytolith Analysis: An Archaeological and Geological Perspective.

[34] Piperno DR (2006). Phytoliths: A Comprehensive Guide for Archaeologists and Paleoecologists.

[35] Piperno DR (1989). The Occurrence of Phytoliths in the Reproductive Structures of Selected Tropical Angiosperms and Their Significance in Tropical Paleoecology, Paleoethnobotany and Systematics.. Rev Palaeobot and Palynol.

[36] Boyde A (1984). Dependence of rate of physical erosion on orientation and density in mineralized tissues.. Anat Embryol.

[37] Richardson RCD (1968). The wear of materials by relatively soft abrasives.. Wear.

[38] Lalueza Fox C, Juan J, Albert RM (1996). Phytolith Analysis on Dental Calculus, Enamel Surface, and Burial Soil: Information About Diet and Paleoenvironment.. Am J Phys Anthropol.

[39] Nystrom P, Phillips-Conroy JE, Jolly CJ (2004). Dental Microwear in Anubis and Hybrid Baboons (*Papio hamadryas, sensu lato*) Living in Awash National Park, Ethiopia.. Am J Phys Anthropol.

[40] Samsonov GV (1968). Handbook of the physicochemical properties of the elements.

[41] Baker G, Jones LHP, Wardrop ID (1959). Cause of wear in sheeps' teeth.. Nature.

[42] Metallic Materials: Conversion of Hardness Values (2003).

[43] Ciochon RL, Piperno DR, Thompson RG, Delson E, Tattersall I, Van Couvering J (1990). Opal phytoliths found on the teeth of the extinct ape *Gigantopithecus blacki*: implications for paleodietary studies.. Paleoanthropology Annuals.

[44] Lucas PW, Teaford MF (1995). Significance of Silica in Leaves to Long-Tailed Macaques (*Macaca fascicularis*).. Folia Primatologica.

[45] Gűgel IL, Grupe G, Kunzelmann K-H (2001). Simulation of Dental Microwear: Characteristic Traces by Opal Phytoliths Give Clues to Ancient Human Dietary Behavior.. Am J Phys Anthropol.

[46] Strömberg CAE (2006). Evolution of hypsodonty in equids: testing of a hypothesis of adaptation.. Paleobiology.

[47] Merceron G, Schulz E, Kordos L, Kaiser TM (2007). Paleoenvironment of *Dryopithecus brancoi* at Rudabánya, Hungary: evidence from dental and meso- and micro-wear analyses of large vegetarian animals.. J Hum Evol.

[48] Sanson GD, Kerr SA, Gross KA (2007). Do silica phytoliths really wear mammalian teeth?. J Arch Sci.

[49] Lalueza Fox C, Pérez-Pérez A (1994). Dietary Information through the Examination of Plant Phytoliths on the Enamel Surface of Human Dentition.. J Arch Sci.

[50] Epstein E (1994). The anomaly of silicon in plant biology.. Proc Natl Acad Sci U S A.

[51] Janis CM, Fortelius M (1988). On the means whereby mammals achieve increased functional durability of their dentitions, with special reference to limiting factors.. Biol Rev.

[52] Hummel J, Findeisen E, Sudekum K-H, Ruf I, Kaiser TM (2010). Another one bites the dust: faecal silica levels in large herbivores correlate with high-crowned teeth.. Proc Royal Soc B.

[53] Dougall HW, Drysdale VM, Glover PE (1964). The Chemical Composition of Kenya Browse and Pasture Herbage.. E Afr Wildlife Journal.

[54] Solounias N, Semprebon GM (2002). Advances in the reconstruction of ungulate ecomorpology with application to early fossil equids.. Am Mus Novitates.

[55] Merceron G, Blondel C, Brunet M, Sen S, Solounias N (2004a). The late Miocene palaeoenvironment of Afghanistan as inferred from dental micro-wear in artiodactyls.. Palaeog Palaeoclimatol Palaeoecol.

[56] Ungar PS, Teaford MF, Glander KE, Pastor RF (1995). Dust accumulation in the canopy: a potential cause of dental microwear in primates.. Am J Phys Anthropol.

[57] Walker A, Hoeck HN, Perez L (1978). Microwear of Mammalian Teeth as an Indicator of Diet.. Science.

[58] Milton K (1981). Food Choice and Digestive Strategies of Two Sympatric Primate Species.. The Am Nat.

[59] Smith CC, Clutton-Brock TH (1977). Feeding Behaviour and Social Organization in Howling Monkeys.. Primate Ecology.

[60] Teaford MF, Glander KE, Norconk MA (1996). Dental Microwear and Diet in a Wild Population of Mantled Howling Monkeys (*Alouatta palliata*).. Adaptive Radiations of Neotropical Primates.

[61] Kelley J (1990). Incisor Microwear and Diet in Three Species of *Colobus*.. Folia Primatologica.

[62] Fashing PJ (2001). Feeding Ecology of Guerezas in the Kakamega Forest, Kenya: The Importance of Moraceae Fruits in Their Diet.. Int J Primatol.

[63] Ungar PS (1995). Fruit Preferences of Four Sympatric Primate Species at Ketambe, Northern Sumatra, Indonesia.. Int J Primatol.

[64] Olejniczak AJ, Grine FE (2006). Assessment of the Accuracy of Dental Enamel Thickness Measurements Using Microfocal X-Ray Computed Tomography.. The Anatomical Record Part A.

[65] Tafforeau P, Boistel R, Boller E, Bravin A, Brunet M (2006). Applications of X- ray synchrotron microtomography for non-destructive 3D studies of paleontological specimens.. Applied Physics A.

[66] Olejniczak AJ, Tafforeau P, Feeney RNM, Martin LB (2008b). Three-dimensional primate molar enamel thickness.. J Hum Evol.

[67] Olejniczak AJ, Smith TM, Skinner MM, Grine FE, Feeney RNM (2008d). Three-dimensional molar enamel distribution and thickness in *Australopithecus* and *Paranthropus*.. Biology Letters.

[68] Felsenstein J (1985). Phylogenies and the comparative method.. Am Nat.

[69] Garland TJ, Harvey PH, Ives AR (1992). Procedures for the analysis of comparative data using phylogenetically independent contrasts.. Syst Biol.

[70] Perelman P, Johnson WE, Roos C, Seuánez HN, Horvath JE (2011). A Molecular Phylogeny of Living Primates.. PLoS Genetics.

[71] Terborgh J, Krebs JR, Clutton-Brock TH (1983). Five New World Primates.

[72] Andriamasimanana M (1994). Ecoethological study of free-ranging aye-ayes (*Daubentonia madagascariensis*) in Madagascar.. Folia Primatologica.

[73] Chalmers NR (1968). Group composition, ecology and daily activities of free living mangabeys in Uganda.. Folia Primatologica.

[74] Bentley-Condit VK (2009). Food choices and habitat use by Tana River yellow baboons (*Papio cynocephalus*): A preliminary report on five years of data.. Am J Primatol.

[75] Wahungu GM (1998). Diet and habitat overlap in two sympatric primate species, the Tana crested mangabey *Cercocebus galeritus* and yellow baboon *Papio cynocephalus*.. Afr J Ecol.

[76] Bamford MK, Albert RM, Cabanes D (2006). Plio-Pleistocene macroplant fossil remains and phytoliths from Lowermost Bed II in the eastern palaeolake margin of Olduvai Gorge, Tanzania.. Quat Internat.

[77] Sterling EJ (1994). Aye-Ayes: Specialists on Structurally Defended Resources.. Folia Primatologica.

[78] Sterling EJ, Dierenfeld ES, Ashbourne CJ, Feistner ATC (1994). Dietary Intake, Food Composition and Nutrient Intake in Wild and Captive Populations of *Daubentonia madagascariensis*.. Folia Primatologica.

[79] Erikson CJ (1994). Tap-Scanning and Extractive Foraging in Aye-Ayes, *Daubentonia madagascariensis*.. Folia Primatologica.

[80] Macho GA, Spears IR (1999). Effects of Loading on the Biochemical Behavior of Molars of *Homo*, *Pan*, and *Pongo*.. Am J Phys Anthropol.

[81] Skogland T (1988). Tooth wear by food limitation and its life history consequences in wild reindeer.. Oikos.

[82] Kojola I, Helle T, Huhta E, Niva A (1998). Foraging conditions, tooth wear and herbivore body reserves.. Oecologia.

[83] King SJ, Arrigo-Nelson SJ, Pochron ST, Semprebon GM, Godfrey LR (2005). Dental senescence in long-lived primate links infant survival to rainfall.. Proc Natl Acad Sci U S A.

[84] Grine FE, Martin LB, Grine FE (1988). Enamel thickness and development in *Australopithecus* and *Paranthropus*.. Evolutionary History of the “Robust” Australopithecines.

[85] Teaford MF, Ungar PS (2000). Diet and the evolution of the earliest human ancestors.. Proc Natl Acad Sci U S A.

[86] Lee-Thorp JA, van der Merwe N, Brain CK (1994). Diet of *Australopithecus robustus* at Swartkrans from stable carbon isotopic analysis.. J Hum Evol.

[87] Sponheimer M, Lee-Thorp JA (1999a). Isotopic evidence for the diet of an early hominid, *Australopithecus africanus*.. Science.

[88] Sponheimer M, Lee-Thorp JA, de Ruiter D, Codron D, Codron J (2005b). Hominins, sedges, and termites: new carbon isotope data from the Sterkfontein valley and Kruger National Park.. J Hum Evol.

[89] van der Merwe NJ, Masao FT, Bamford MK (2008). Isotopic evidence for contrasting diets of early hominins *Homo habilis* and *Australopithecus boisei*.. S Afr J Sci.

[90] Teaford MF, Jablonski NG (1993). Dental microwear and diet in extant and extinct *Theropithecus*: preliminary analysis.. *Theropithecus*: The Rise and Fall of a Primate Genus.

[91] Kuroda S, Nishihara T, Suzuki S, Oko RA, McGrew WC, Nishida T, Marchant LA (1996). Sympatric chimpanzees and gorillas in the Ndoki Forest, Congo.. Great Ape Societies.

[92] Peters CR, O'Brien EM (1981). The early hominid plant-food niche: insights from an analysis of plant exploitation by *Homo*, *Pan*, and *Papio* in Eastern and Southern Africa.. Curr Anthropol.

[93] Tutin CEG, Ham RM, White LJT, Harrison MJS (1997). The Primate Community of the Lopé Reserve, Gabon: Diets, Responses to Fruit Scarcity, and Effects of Biomass.. Am J Primatol.

[94] Wright BW (2005). Craniodental biomechanics and dietary toughness in the genus *Cebus*.. J Hum Evol.

[95] Hatley T, Kappelman J (1980). Bears, pigs, and Plio-Pleistocene hominids: a case for the exploitation of below-ground resources.. Human Ecology.

[96] Dominy NJ, Vogel ER, Yeakel JD, Constantino P, Lucas PW (2008). Mechanical Properties of Plant Underground Storage Organs and Implications for Dietary Models of Early Hominins.. Evolutionary Biology.

[97] Conklin-Brittain NL, Wrangham RW, Smith CC, Ungar PS, Teaford MF (2002). A two-stage model of increased dietary quality in early hominid evolution: the role of fiber.. Human Diet: Its Origin and Evolution.

[98] Milton K (1999). A Hypothesis to Explain the Role of Meat-Eating in Human Evolution.. Evol Anthropol.

[99] Peters CR, Timberlake J, Kativu S (1999). African wild plants with rootstocks reported to be eaten raw: the monocotyledons, Part IV.. African Plants: Biodiversity, Taxonomy and Uses.

[100] Wrangham RW, Conklin NL, Chapman CA, Hunt KD (1991). The significance of fibrous foods for Kibale Forest chimpanzees.. Phil Trans R Soc Lond B.

[101] Lanning FC (1965). Silica and Calcium Deposition in the Tissues of Certain Plants.. Advancing Frontiers of Plant Sciences.

[102] Martin LB (1983). The relationships of the later Miocene Hominoidea [PhD].

[103] Eck GG, Jablonski NG, Eck GG, Jablonski NG, Leakey MD (1987). The skull of *Theropithecus brumpti* compared with those of other species of the genus *Theropithecus*.. Les faunes Plio-Pleistocene de la Vallée de L'Omo (Ethiopie) 3. Cercopithecidae de la formation de Shungura.

[104] Mau M, Sudekum K-H, Achim J, Sliwa A, Kaiser TM (2009). Saliva of the Graminivorous *Theropithecus gelada* Lacks Proline-Rich Proteins.. Am J Primatol.

[105] McNaughton SJ, Tarrants JL, McNaughton MM, Davis RH (1985). Silica as a Defense Against Herbivory and a Growth Promotor in African Grasses.. Ecology.

[106] Constantino P, Wood B (2007). The Evolution of *Zinjanthropus boisei*.. Evol Anthropol.

[107] Tattersall I (1982). The Primates of Madagascar.

[108] Dumont ER, Ryan TM, Godfrey LR (2011). The *Hadropithecus* conundrum reconsidered, with implications for interpreting diet in fossil hominins.. Proc R Soc B published online.

[109] Peters CR, Vogel JC (2005). Africa's wild C_4_ plant foods and possible early hominid diets.. J Hum Evol.

[110] Peters CR, O'Brien EM, Drummond RB (1992). Edible wild plants of sub-Saharan Africa.

[111] Shumway CA (1999). Forgotten Waters: freshwater and marine ecosystems in Africa.

[112] Shipman P, Harris JM, Grine FE (1988). Habitat preference and paleoecology of *Australopithecus boisei* in Eastern Africa.. Evolutionary History of the “Robust” Australopithecines.

[113] Reed KE (1997). Early hominid evolution and ecological change through the African Plio-Pleistocene.. J Hum Evol.

[114] Bowes G, Rao SK, Estavillo GM, Reiskind JB (2002). C_4_ mechanisms in aquatic angiosperms: comparisons with terrestrial C_4_ systems.. Functional Plant Biology.

[115] Levin NE, Cerling TE, Passey BH, Harris JM, Ehleringer JR (2006). A stable isotope aridity index for terrestrial environments.. Proc Natl Acad Sci U S A.

