# Supplementary material for: Abrasive, Silica Phytoliths and the Evolution of Thick Molar Enamel in Primates, with Implications for the Diet of Paranthropus boisei
Source: PLoS One. 2011 Dec 7;6(12):e28379. doi: 10.1371/journal.pone.0028379 (PMC3233556; doi:10.1371/journal.pone.0028379)
Supplement: Text S1 — Relative enamel thickness (RET) values. Additional information on the methodology used in the literature to obtain these values. (DOC) [file pone.0028379.s001.doc]

**Text S1**

**Relative enamel thickness (RET) values**

In 1983, Martin found that the volume of enamel tissue on a tooth provided the most useful method of quantifying its molar enamel thickness.^1^ He proposed slicing a thin section of a specimen molar vertically through the tips of the mesial cusps, then viewing it under a scanning electron microscope and taking the following measurements:

1. The area of the enamel cap (= c)
2. The length of the enamel-dentine junction (EDJ) (= e)

Dividing the area of the enamel cap (c) by the length of the EDJ (e) yields a value termed the “average enamel thicknesss” (AET = c/e). As tooth size is known to scale isometrically with body size, resulting in higher volumes of enamel on the molars of larger-bodied animals, it was necessary to control for body size in order to make comparisons between primates, including fossil primates, for which body size is not known. Martin found that the body size of an animal is closely correlated with the combined area of dentine and pulp in the same section on which enamel thickness was measured.^2^ He therefore combined the value of average enamel thickness with the sum of the areas of the dentine and pulp, in the following formula:

(c/e x 100)/√b

where: c = area of the enamel cap;

e = length of the enamel-dentine junction (EDJ) over which the enamel formed;

b = combined area of dentine and pulp in the same section.

This formula produces a dimensionless index termed the “relative enamel thickness” (RET).

The sources for the RETs used in our sample are found in Table S1.

_____________________

^1^ Martin LB (1983) The relationships of the later Miocene Hominoidea. [PhD] London: University College London.

^2^ Martin LB (1985) Significance of enamel thickness in hominoid evolution. Nature 314: 260-263.
