# Supplementary material for: Abrasive, Silica Phytoliths and the Evolution of Thick Molar Enamel in Primates, with Implications for the Diet of Paranthropus boisei
Source: PLoS One. 2011 Dec 7;6(12):e28379. doi: 10.1371/journal.pone.0028379 (PMC3233556; doi:10.1371/journal.pone.0028379)
Supplement: Text S2 — Dietary Criteria. Additional information on the criteria used to select the primate species used in our sample. (DOC) [file pone.0028379.s002.doc]

**Text S2**

**Dietary Studies**

We gathered dietary data from at least 2 and up to 6 studies representing differing habitats for each primate in our sample. For our model, the studies needed to provide proportional percentages for each plant food species and/or family consumed, along with the total percentage of leaves in the diet. For *Cercocebus torquatus* only one study provided the proportional data required. To reach our desired minimum of two studies per primate, we added a study of *Cercocebus galeritus.* *C. torquatus* and *C. g. galeritus* are very closely related, both belonging to the *C. torquatus* group of monkeys, which inhabit riverine forest almost exclusively.^1^ In this regard, Homewood’s *C. g. galeritus* study area better reflects the more usual habitat and consequent diet of *C. torquatus* than does Mitani’s study area for *C. torquatus*, which is depauperate in the figs and palm trees typical of their habitat in other areas.^2^

Both studies of *Cebus capucinus* that had the requisite dietary data came from the same type of habitat, the tropical dry forests of Costa Rica, yet this primate is well known to inhabit more mesic habitats as well.^3^ We therefore added a study of *Cebus olivaceous*, a closely related primate, from a mesic habitat.^4^ The dietary preferences of *C. capucinus* and *C. olivaceous* have been shown to overlap significantly.^3^

Dietary data for all other primates in the sample were drawn solely from studies of the species listed.

All dietary data came from direct observations of either selected individuals or groups, or both, usually through dusk-to-dawn follows and focal animal scan sampling methods standard for the field.^5^ No study used fecal analysis as the sole data source for diet composition, as such analysis is known to underestimate—or even miss altogether—the consumption of certain plant parts.^6^ However, several studies used a combination of direct observations, collection of trail remains, and fecal analyses to arrive at diet composition.

_______________________

^1^ Homewood KM (1978) Feeding strategy of the Tana mangabey (*Cercocebus galeritus galeritus*) (Mammalia: Primates). J Zool Lond 186: 375-391.

^2^ Mitani M (1989) *Cercocebus torquatus*: adaptive feeding and ranging behaviors related to seasonal fluctuations of food resources in the tropical rain forest of south-western Cameroon. Primates 30: 307-323.

^3^ Fragaszy DM, Visalberghi E, Fedigan LM (2004) The Complete Capuchin: The Biology of the Genus *Cebus*. Cambridge: Cambridge University Press. 339 p.

^4^ Robinson JG (1984) Diurnal Variation in Foraging and Diet in Wedge-Capped Capuchin *Cebus* *olivaceous*. Folia Primatologica 43: 216-228.

^5^ Altmann J (1974) Observational study of behavior. Sampling methods. Behaviour 49: 227-267.

^6^ Tutin CEG, Fernandez M (1993) Composition of the diet of chimpanzees and comparisons with that of sympatric lowland gorillas in the Lopé Reserve, Gabon. Am J Primatol 30: 195-211.
