# Supplementary figures and images for: Abrasive, Silica Phytoliths and the Evolution of Thick Molar Enamel in Primates, with Implications for the Diet of Paranthropus boisei
Source: PLoS One. 2011 Dec 7;6(12):e28379. doi: 10.1371/journal.pone.0028379 (PMC3233556; doi:10.1371/journal.pone.0028379)

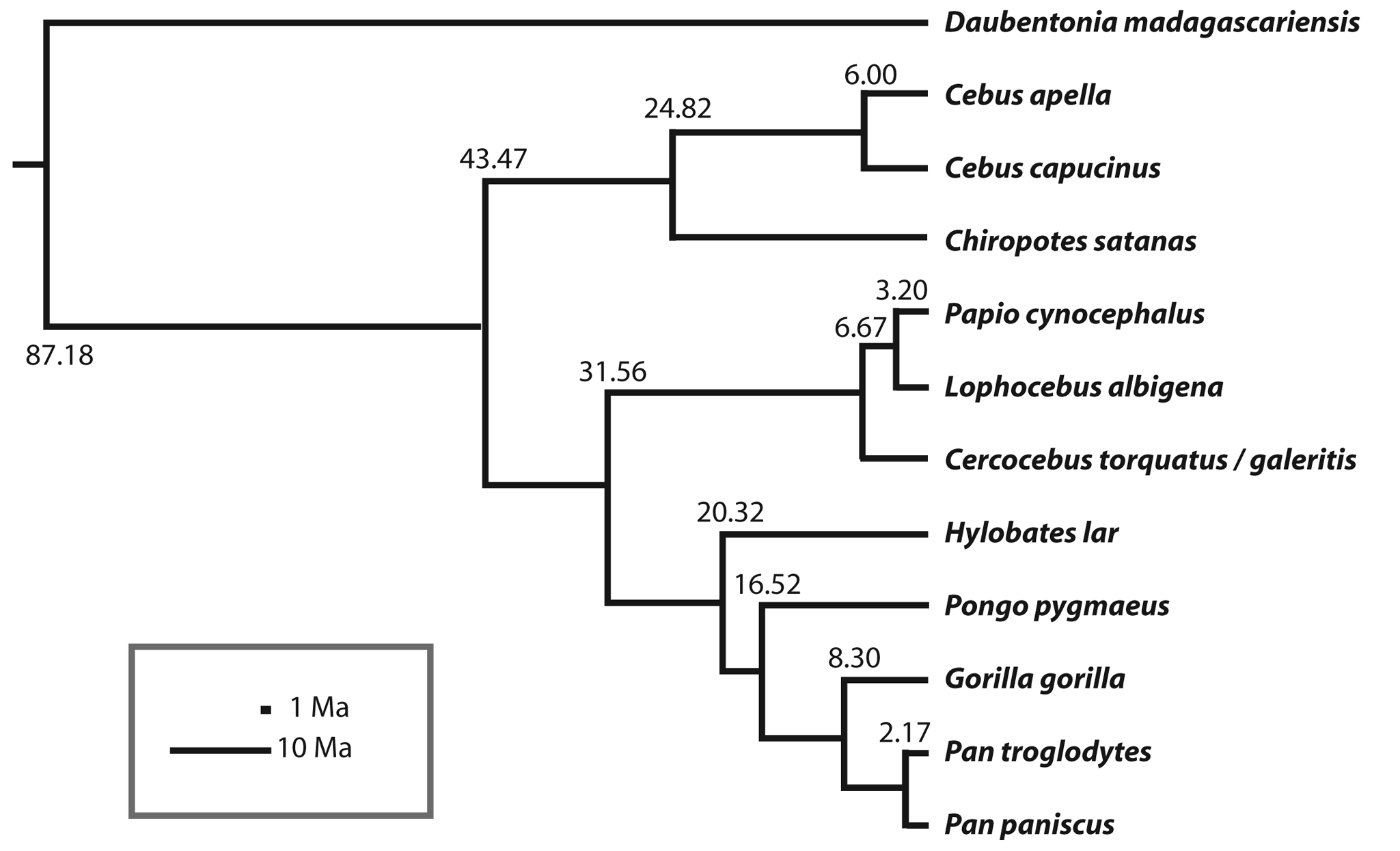

Supplement: Figure S1 — Phylogeny of the species included in the study with ages of splits indicated in millions of years (data from Perelman et al., 2011 [70] ). The phylogeny (Figure S1) of the twelve primate species was transformed into eleven phylogentically independent contrasts following Garland et al.'s [69] application of Felsenstein's method [68]. The phylogenetically independent contrasts used in some of the analyses are as follows: Contrast 1, Cebus apella versus Cebus capucinus; Contrast 2, Chiropotes versus the Cebus clade; Contrast 3, Pan paniscus versus Pan troglodytes; Contrast 4, Gorilla versus the Pan-Homo clade; Contrast 5, Pongo versus the African ape clade; Contrast 6, Hylobates lar versus the great ape clade; Contrast 7, Lophoccebus albigena versus Papio cynocephalus; Contrast 8, Clade of Lophocebus plus Papio versus the amalgam of Cercocebus torquatus/C. galeritis; Contrast 9, Old World monkeys versus Hominoids; Contrast 10, Platyrrhines versus Catarrhines; Contrast 11, Daubentonia madagascariensis versus Anthropoids. (TIF) [file pone.0028379.s003.tif]
