# Supplementary material for: Abrasive, Silica Phytoliths and the Evolution of Thick Molar Enamel in Primates, with Implications for the Diet of Paranthropus boisei
Source: PLoS One. 2011 Dec 7;6(12):e28379. doi: 10.1371/journal.pone.0028379 (PMC3233556; doi:10.1371/journal.pone.0028379)
Supplement: Table S1 — Dietary studies from which data was obtained for each primate species in the sample, along with sources of their RET values. (DOC) [file pone.0028379.s004.doc]

Table S1. Dietary studies from which data was obtained for each primate species in the sample, along with sources of their RET values.

| **Primate Species** | **RET** | **Dietary Studies** |
| --- | --- | --- |
|  |  |  |
| *Cebus apella* | 19.57^b^ | Brown & Zunino, 1990^1^; Janson, 1985^2^; Spironello, 2001^3^ |
| *Cebus capucinus* | 15.13^b^ | Williams & Vaughn, 2001^4^; Chapman & Fedigan, 1990^5^; Robinson, 1984^6^ *(C. olivaceus)* |
| *Cercocebus torquatus* | 12.89^b^ | Mitani, 1991^7^; Homewood, 1978^8^ *(C. galeritus galeritus)* |
| *Chiropotes satanas* | 9.54^d^ | van Roosmalen et al., 1988^9^; Port-Carvalho & Ferrari, 2004^10^; Peetz, 2001^11^ |
| *Daubentonia madagascariensis* | 21.68^a^ | Andriamasimanana, 1994^12^; Sterling, 1994^13^ and Sterling et al., 1994^14^ |
| *Gorilla* spp. | 9.66^a^ | Doran-Sheehy et al., 2006^15^; Yamagiwa et al., 2005^16^; Watts, 1984^17^ |
| *Hylobates lar* | 11.09^a^ | Ungar, 1995^18^; Bartlett,1999^19^ and 2009^20^; MacKinnon & MacKinnon, 1980^21^ |
| *Lophocebus albigena* | 16.85^b^ | Waser , 1975^22^; Waser, 1977^23^; Freeland, 1979^24^; Ham, 1994^25^; Poulsen et al., 2001^26^ |
| *Pan paniscus* | 14.0^c^ | Kano, 1983^27^; Badrian & Malenky, 1984^28^ |
| *Pan troglodytes* | 11.60^a^ | Nishida & Uehara, 1983^29^; Ghiglieri, 1984^30^; Newton-Fisher, 1999^31^; Emery Thompson, 2005: Appendices B(1), B(2), (B3)^32^; Morgan & Sanz, 2006^33^ |
| *Papio cynocephalus* | 16.11^a^ | Bentley-Condit, 2009^34^; Post, 1982^35^ |
| *Pongo pygmaeus* | 15.33^b^ | Hamilton & Galdikas, 1994^36^; Ungar, 1995^18^; MacKinnon, 1974^37^; Galdikas, 1978^38^ |

^a^ Shellis *et al*, 1998^39^; ^b^ Dumont, 1995^40^; ^c^ Smith et al, 2003^41^; ^d^ Martin et al, 2003^42^*.*

^1^ Brown AD, Zunino GE (1990) Dietary variability in *Cebus apella* in extreme habitats: evidence for adaptability. Folia Primatologica 54: 177-186.

^2^ Janson CH (1985) Aggressive competition and individual food consumption in wild brown capuchin monkeys (*Cebus apella*). Behav Ecol Sociobiol 18: 125-138.

^3^ Spironello WR (2001) The Brown Capuchin Monkey *(Cebus apella)*: Ecology and Home Range Requirements in Central Amazonia. In: Bierregaard J, Richard O, Lovejoy TE, Mesquita RCG, editors. Lessons from Amazonia: The Ecology and Conservation of a Fragmented Forest. New Haven: Yale University Press. pp. 271- 283.

^4^ Williams HE, Vaughn C (2001) White-faced monkey (*Cebus capucinus*) ecology and management in neotropical agricultural landscapes during the dry season. Revista de Biologia Tropical 49: 1199-1206.

^5^ Chapman CA, Fedigan LM (1990) Dietary differences between neighboring *Cebus* *capucinus* groups: local traditions, food availability or responses to food profitability? Folia Primatologica 54: 177-186.

^6^ Robinson JG (1984) Diurnal Variation in Foraging and Diet in Wedge-Capped Capuchin *Cebus olivaceus*. Folia Primatologica 43: 216-228.

^7^ Mitani M (1991) Niche Overlap and Polyspecific Associations Among Sympatric

Cercopithecids in the Campo Animal Reserve, southwestern Cameroon. Primates 32: 137-151.

^8^ Homewood KM (1978) Feeding strategy of the Tana mangabey (Cercocebus galeritus galeritus) (Mammalia: Primates) J Zool Lond 186: 375-391.

^9^ van Roosmalen MGM, Mittermeier RA, Fleagle JG (1988) Diet of the Northern Bearded Saki (*Chiropotes satanas chiropotes*): A Neotropical Seed Predator. Am J Primatol 14: 11-35.

^10^ Port-Carvalho M, Ferrari SF (2004) Occurrence and Diet of the Black Bearded Saki

(*Chiropotes satanas satanas*) in the Fragmented Landscape of Western Maranhão, Brazil. Neotropical Primates 12: 17-21.

^11^ Peetz A (2001) Ecology and Social Organization of the Bearded Saki *Chiropotes satanas chiropotes* (Primates: Pitheciinae) in Venezuela. In: Schuchmann K-L, ed. Ecological Monographs, Vol. 1. Bonn: Society of Tropical Ecology. 170 p.

^12^ Andriamasimanana M (1994) Ecoethological Study of Free-Ranging Aye-Ayes

(*Daubentonia madagascariensis*) in Madagascar. Folia Primatologica 62: 37-45.

^13^ Sterling EJ (1994) Aye-Ayes: Specialists on Structurally Defended Resources. Folia Primatologica 62: 142-154.

^14^ Sterling EJ, Dierenfeld ES, Ashbourne CJ, Feistner ATC (1994) Dietary Intake, Food Composition and Nutrient Intake in Wild and Captive Populations of *Daubentonia madagascariensis.* Folia Primatologica 62: 115-124.

^15^ Doran-Sheehy DM, Shah NF, Heimbauer LA (2006) Sympatric western gorilla and

mangabey diet: re-examination of ape and monkey foraging strategies. In: Hohmann G, Robbins MM, Boesch C, editors. Feeding Ecology in Apes and Other Primates. New York: Cambridge University Press. pp. 49-72.

^16^ Yamagiwa J, Basabose AK, Kaleme K, Yumoto T (2005) Diet of Grauer’s Gorillas in the Montane Forest of Kahuzi, Democratic Republic of Congo. Int J Primatol 26: 1345-1373.

^17^ Watts DP (1984) Composition and Variability of Mountain Gorilla Diets in the central Virungas. Am J Primatol **7:** 323-356.

^18^ Ungar PS (1995) Fruit Preferences of Four Sympatric Primate Species at Ketambe, Northern Sumatra, Indonesia. Int J Primatol 16: 221-245.

^19^ Bartlett TQ (1999) Feeding and ranging behavior of the white-handed gibbon *(Hylobates lar)* in Khao Yai National Park, Thailand. [PhD] St. Louis, MO: Washington University.

^20^ Bartlett TQ (2009) The Gibbons of Khao Yai. Upper Saddle River, NJ: Pearson Education. 170 p.

^21^ MacKinnon JR, MacKinnon KS (1980) Niche Differentiation in a Primate Community. In: Chivers DJ, editor. Malayan Forest Primates: Ten Years’ Study in Tropical Rain Forest. New York: Plenum Press. pp. 167-190.

^22^ Waser P (1975) Monthly variations in feeding and activity patterns of the mangabey, *Cercocebus albigena* (Lydekker). Afr J Ecol 13: 249-263.

^23^ Waser P (1977) Feeding, Ranging and Group Size in the Mangabey *Cercocebus albigena*. In: Clutton-Brock TH, editor. Primate Ecology: Studies of feeding and ranging in lemurs, monkeys, and apes. New York: Academic Press. pp. 183-222.

^24^ Freeland WJ (1979) Mangabey (*Cercocebus albigena*): social organization and

population density in relation to food use and availability. Folia Primatologica 32: 108-124.

^25^ Ham RM (1994) Behavior and Ecology of Grey-Cheeked Mangabeys *(Cercocebus*

*albigena)* in the Lopé Reserve, Gabon. [PhD] Stirling: Stirling University.

^26^ Poulsen CJ, Clark CJ, Smith TB (2001) Seasonal Variation in the Feeding Ecology of the Grey-Cheeked Mangabey (*Lophocebus albigena*) in Cameroon. Am J Primatol 54: 91-105.

^27^ Kano T (1983) An Ecological Study of the Pygmy Chimpanzees. Int J Primatol 4: 1-31.

^28^ Badrian N, Malenky RK (1984) Feeding Ecology of *Pan paniscus* in the Lomako Forest, Zaire. In: Susman RL, editor. The Pygmy Chimpanzee. New York and London: Plenum Press. pp. 233-299.

^29^ Nishida T, Uehara S (1983) Natural Diet of Chimpanzees (*Pan troglodytes schweinfurthii*): Long-term Record from the Mahale Mountains, Tanzania. Afr Study Monograph 3: 109-130.

^30^ Ghiglieri MP (1984a) The Chimpanzees of Kibale Forest: A Field Study of Ecology and Social Structure. New York: Columbia University Press. 226 p.

^31^ Newton-Fisher NE (1999) The diet of chimpanzees in the Budongo Forest Reserve, Uganda. Afr J Ecol 37: 344-354.

^32^ Emery Thompson M (2005) Endocrinology and Ecology of Wild Female Chimpanzee Reproduction. [PhD] Cambridge: Harvard University.

^33^ Morgan D, Sanz C, (2006) Chimpanzee feeding ecology and comparisons with sympatric gorillas in the Goualougo Triangle, Republic of Congo. In: Hohmann G, Robbins MM, Boesch C, editors. Feeding Ecology in Apes and Other Primates. Cambridge: Cambridge University Press. pp. 97-122.

^34^ Bentley-Condit VK (2009) Food Choices and Habitat Use by the Tana River Yellow Baboons (*Papio cynocephalus*): A Preliminary Report on Five Years of Data. Am J Primatol 71: 432-436.

^35^ Post DG (1982) Feeding Behavior of Yellow Baboons (*Papio cynocephalus*) in the Amboseli National Park, Kenya. Int J Primatol 3: 403-430.

^36^ Hamilton RA, Galdikas BMF (1994) A Preliminary Study of Food Selection by the Orangutan in Relation to Plant Quality. Primates 35: 255-263.

^18^ Ungar PS (1995) Fruit Preferences of Four Sympatric Primate Species at Ketambe, Northern Sumatra, Indonesia. Int J Primatol 16: 221-245.

^37^ MacKinnon J (1974) The Behaviour and Ecology of Wild Orang-Utans (*Pongo pygmaeus*). Animal Behaviour 22: 3-74.

^38^ Galdikas BMF (1978) Orangutan Adaptation at Tanjung Puting Reserve, Central Borneo. [PhD] Los Angeles: University of California.

^39^ Shellis RP, Beynon AD, Reid DJ, Hiiemae KM (1998) Variations in molar enamel thickness among primates. J Hum Evol 35: 507-522.

^40^ Dumont ER (1995) Enamel Thickness and Dietary Adaptation Among Extant Primates and Chiropterans. J Mammal 76: 1127-1136.

^41^ Smith TM, Martin LB, Leakey MG (2003) Enamel thickness, microstructure and development in *Afropithecus turkanensis*. J Hum Evol 44: 283-306.

^42^ Martin LB, Olejniczak AJ, Maas MC (2003) Enamel thickness and microstructure in pitheciin primates, with comments on dietary adaptations of the middle Miocene hominoid *Kenyapithecus*. J Hum Evol*.* 45: 351-367.
