# Supplementary material for: Abrasive, Silica Phytoliths and the Evolution of Thick Molar Enamel in Primates, with Implications for the Diet of Paranthropus boisei
Source: PLoS One. 2011 Dec 7;6(12):e28379. doi: 10.1371/journal.pone.0028379 (PMC3233556; doi:10.1371/journal.pone.0028379)
Supplement: Table S2 — Transformed data for the phylogenetically independent contrasts. (DOC) [file pone.0028379.s005.doc]

**Table S2**. Transformed data for the phylogentically independent contrasts.

| Contrast | RET_  diff | Phytolith_  A_diff | Phytolith_B  _diff | %_Leaves  _diff | SQRT  (SumBrachL) |
| --- | --- | --- | --- | --- | --- |
| 1 | 4.44 | 21.24 | 21.69 | -1.97 | 3.464 |
| 2 | 7.81 | 28.48 | 18.18 | -0.74 | 6.606 |
| 3 | 2.40 | 12.78 | 13.86 | 0.73 | 2.083 |
| 4 | 3.14 | 27.88 | 28.30 | -31.33 | 3.799 |
| 5 | 4.10 | -13.99 | -20.91 | -17.85 | 4.974 |
| 6 | 2.19 | -3.07 | -3.69 | 6.00 | 4.911 |
| 7 | 0.75 | -28.00 | -40.32 | 1.50 | 2.530 |
| 8 | 3.59 | 22.84 | 18.75 | -2.59 | 3.184 |
| 9 | 2.50 | -5.77 | -5.13 | -17.13 | 6.011 |
| 10 | 0.01 | -4.53 | -6.35 | -3.09 | 5.528 |
| 11 | 8.23 | 20.64 | 3.53 | -7.19 | 11.441 |

In Table S2, each column shows the difference (diff) in values in the raw data for the contrast, calculated as the value for taxon or operational taxonomic unit (OUT) with the higher RET minus the value for the OTU with the lower RET^1^. The resultant values shown here are unscaled by branch length.

Values for SQRT(SumBranchL) are square root of the summed branch length (in millions of years) between the nodes under consideration. To adjust for branch length, RET_diff, Phtyolith_A_diff, Pytolith_B_diff, and %_Leaves_diff are divided by SQRT(SumBranchL) (adjusted values not shown).

^1^ Nunn C, Barton R (2001) Comparative methods for studying primate adaptation and allometry. *Evolutionary Anthropology* 10: 81-98.
