# Supplementary material for: Abrasive, Silica Phytoliths and the Evolution of Thick Molar Enamel in Primates, with Implications for the Diet of Paranthropus boisei
Source: PLoS One. 2011 Dec 7;6(12):e28379. doi: 10.1371/journal.pone.0028379 (PMC3233556; doi:10.1371/journal.pone.0028379)
Supplement: Table S3 — Multiple regression on raw data to predict RET from dietary variables. (DOC) [file pone.0028379.s006.doc]

**Table S3**. Multiple regression on raw data to predict RET from dietary variables.

Summary of Fit

| RSquare | 0.871457 |
| --- | --- |
| RSquare Adj | 0.823253 |
| Root Mean Square Error | 1.591962 |
| Mean of Response | 14.47083 |
| Observations | 12 |

Analysis of Variance

| Source | DF | Sum of Squares | Mean Square | F Ratio |
| --- | --- | --- | --- | --- |
| Model | 3 | 137.45214 | 45.8174 | 18.0786 |
| Error | 8 | 20.27475 | 2.5343 | **Prob > F** |
| C. Total | 11 | 157.72689 |  | 0.0006 |

Parameter Estimates

| Term |  | Estimate | Std Error | t Ratio | Prob>|t| |
| --- | --- | --- | --- | --- | --- |
| Intercept |  | 7.414629 | 1.986977 | 3.73 | 0.0058 |
| Phytolith load A |  | 0.4515076 | 0.081589 | 5.53 | 0.0006 |
| Phytolith load B |  | -0.357871 | 0.078073 | -4.58 | 0.0018 |
| % leaves eaten |  | -0.081616 | 0.037286 | -2.19 | 0.0600 |
