# Supplementary material for: Abrasive, Silica Phytoliths and the Evolution of Thick Molar Enamel in Primates, with Implications for the Diet of Paranthropus boisei
Source: PLoS One. 2011 Dec 7;6(12):e28379. doi: 10.1371/journal.pone.0028379 (PMC3233556; doi:10.1371/journal.pone.0028379)
Supplement: Table S4 — Multiple regression on data transformed into phylogenetically independent contrasts. These contrasts predict the difference in RET from differences in the dietary variables, unscaled by branch length (time). (DOC) [file pone.0028379.s007.doc]

**Table S4**. Multiple regression on data transformed into phylogenetically independent contrasts to predict the difference in RET (RET_diff) from differences in the dietary variables. These data are unscaled by branch length (time).

Summary of Fit

| RSquare | 0.764349 |
| --- | --- |
| RSquare Adj | 0.663356 |
| Root Mean Square Error | 1.49013 |
| Mean of Response | 3.56 |
| Observations | 11 |

Analysis of Variance

| **Source** | **DF** | **Sum of Squares** | **Mean Square** | **F Ratio** |
| --- | --- | --- | --- | --- |
| Model | 3 | 50.415993 | 16.8053 | 7.5683 |
| Error | 7 | 15.543407 | 2.2205 | **Prob > F** |
| C. Total | 10 | 65.959400 |  | 0.0134 |

Parameter Estimates

| **Term** |  | **Estimate** | **Std Error** | **t Ratio** | **Prob>|t|** |
| --- | --- | --- | --- | --- | --- |
| Intercept |  | 1.630187 | 0.686727 | 2.37 | 0.0493 |
| Phyto_A_diff |  | 0.3449093 | 0.082385 | 4.19 | 0.0041 |
| Phyto_B_diff |  | -0.254315 | 0.076874 | -3.31 | 0.0130 |
| %_Leaves_diff |  | -0.016975 | 0.04382 | -0.39 | 0.7100 |
