# Supplementary material for: Abrasive, Silica Phytoliths and the Evolution of Thick Molar Enamel in Primates, with Implications for the Diet of Paranthropus boisei
Source: PLoS One. 2011 Dec 7;6(12):e28379. doi: 10.1371/journal.pone.0028379 (PMC3233556; doi:10.1371/journal.pone.0028379)
Supplement: Table S5 — Multiple regression on data transformed into phylogenetically independent contrasts, scaled by time. (DOC) [file pone.0028379.s008.doc]

**Table S5**. Multiple regression on data transformed into phylogenetically independent contrasts, scaled by time.

Summary of Fit

| RSquare | 0.660913 |
| --- | --- |
| RSquare Adj | 0.51559 |
| Root Mean Square Error | 0.29161 |
| Mean of Response | 0.75216 |
| Observations | 11 |

Analysis of Variance

| **Source** | **DF** | **Sum of Squares** | **Mean Square** | **F Ratio** |
| --- | --- | --- | --- | --- |
| Model | 3 | 1.1602062 | 0.386735 | 4.5479 |
| Error | 7 | 0.5952534 | 0.085036 | **Prob > F** |
| C. Total | 10 | 1.7554596 |  | 0.0453 |

Parameter Estimates

| **Term** |  | **Estimate** | **Std Error** | **t Ratio** | **Prob>|t|** |
| --- | --- | --- | --- | --- | --- |
| Intercept |  | 0.5196944 | 0.149315 | 3.48 | 0.0103 |
| Phytolith_A_diff_scaled |  | 0.2234585 | 0.104141 | 2.15 | 0.0690 |
| Phytolith_B_diff_scaled |  | -0.140342 | 0.086577 | -1.62 | 0.1490 |
| %leaves_diff_scaled |  | -0.0166801 | 0.036016 | 0.46 | 0.6573 |

Here time is represented by the square root of the time represented by the sum of branch lengths between the nodes under consideration. The scaled contrasts were then used to predict difference in scaled RET (RET_diff_scaled). Controlling for time diminishes the value of R^2^, but the model remains significant at p < 0.05.
