# Supplementary material for: Abrasive, Silica Phytoliths and the Evolution of Thick Molar Enamel in Primates, with Implications for the Diet of Paranthropus boisei
Source: PLoS One. 2011 Dec 7;6(12):e28379. doi: 10.1371/journal.pone.0028379 (PMC3233556; doi:10.1371/journal.pone.0028379)
Supplement: Table S6 — Correlations between the raw variables. (DOC) [file pone.0028379.s009.doc]

**Table S6**. Correlations between the raw variables.

|  | **RET** | **Phytolith**  **Load A** | **Phytolith**  **Load B** | **% Leaves Eaten** |
| --- | --- | --- | --- | --- |
| RET | 1.0000  (p ≤ 0.0000) |  |  |  |
| Phytolith Load A | 0.4667  (p = 0.1261) | 1.0000  (p ≤ 0.0000) |  |  |
| Phytolith Load B | 0.1205  (p = 0.7091) | 0.9149  (p < 0.0001) | 1.0000  (p ≤ 0.0000) |  |
| % Leaves Eaten | -0.5861  (p = 0.0452) | -0.0531  (p = 0.8698) | 0.1152  (p = 0.7213) | 1.0000  (p ≤ 0.0000) |
