# Supplementary material for: Abrasive, Silica Phytoliths and the Evolution of Thick Molar Enamel in Primates, with Implications for the Diet of Paranthropus boisei
Source: PLoS One. 2011 Dec 7;6(12):e28379. doi: 10.1371/journal.pone.0028379 (PMC3233556; doi:10.1371/journal.pone.0028379)
Supplement: Table S7 — Correlations between variables transformed into phylogenetically independent contrasts, unscaled by time. (DOC) [file pone.0028379.s010.doc]

**Table S7**. Correlations between variables transformed into phylogenetically independent contrasts, unscaled by time.

|  | **RET_diff** | **Phytolith_A_diff** | **Phytolith_B_diff** | **%_Leaves_**  **diff** | **SQRT(SumBrachL)** |
| --- | --- | --- | --- | --- | --- |
| RET_diff | 1.0000  (p ≤ 0.0000) |  |  |  |  |
| Phyto_A_diff | 0.6287  (p = 0.0383) | 1.0000  (p ≤ 0.0000) |  |  |  |
| Phyto_B_diff | 0.4174  (p = 0.2015) | 0.9534  (p < 0.0001) | 1.0000  (p ≤ 0.0000) |  |  |
| %_Leaves_diff | -0.0677  (p = 0.8433) | -0.1461  (p = 0.6682) | -0.1861  (p = 0.5837) | 1.0000  (p ≤ 0.0000) |  |
| SQRT(SumBrachL) | 0.6539  (p = 0.0291) | 0.2000  (p = 0.5554) | 0.0061  (p = 0.9857) | -0.1056  (p = 0.7572) | 1.0000  (p ≤ 0.0000) |

Time, gauged by the square root of summed branch lengths between the nodes under consideration [SQRT(SumBranchL)], is also included in the table. It shows a reasonable correlation with the differences in RET (RET_diff), but not with any of the dietary variables.
