# Supplementary material for: Abrasive, Silica Phytoliths and the Evolution of Thick Molar Enamel in Primates, with Implications for the Diet of Paranthropus boisei
Source: PLoS One. 2011 Dec 7;6(12):e28379. doi: 10.1371/journal.pone.0028379 (PMC3233556; doi:10.1371/journal.pone.0028379)
Supplement: Table S8 — Correlations between variables transformed into phylogenetically independent contrasts scaled by time. (DOC) [file pone.0028379.s011.doc]

**Table S8**. Correlations between variables transformed into phylogenetically independent contrasts scaled by time.

|  | **RET_**  **diff_t** | **Phytolith_A**  **_diff_t** | **Phytolith_B**  **_diff_t** | **%leaves**  **_diff_t** |
| --- | --- | --- | --- | --- |
| RET_diff_t | 1.0000  (p ≤ 0.0000) |  |  |  |
| Phytolith_A_diff_t | 0.7163  (p = 0.0131) | 1.0000  (p ≤ 0.0000) |  |  |
| Phytolith_B_diff_t | 0.6481  (p = 0.0311) | 0.9872  (p < 0.0001) | 1.0000  (p ≤ 0.0000) |  |
| %leaves_diff_t | -0.0564  (p = 0.8691) | -0.2711  (p = 0.4200) | -0.2850  (p = 0.3956) | 1.0000  (p ≤ 0.0000) |
